# Fetal Hypoglycemia Induced by Placental *SLC2A3*-RNA Interference Alters Fetal Pancreas Development and Transcriptome at Mid-Gestation

**DOI:** 10.3390/ijms25094780

**Published:** 2024-04-27

**Authors:** Victoria C. Kennedy, Cameron S. Lynch, Amelia R. Tanner, Quinton A. Winger, Ahmed Gad, Paul J. Rozance, Russell V. Anthony

**Affiliations:** 1 College of Veterinary Medicine, Colorado State University, Fort Collins, CO 80523, USA; tori.kennedy@colostate.edu (V.C.K.); cameronlynch553@gmail.com (C.S.L.); amelia.tanner@cuanschutz.edu (A.R.T.); quinton.winger@colostate.edu (Q.A.W.); ahmed.gad@colostate.edu (A.G.); 2 University of Colorado School of Medicine, Aurora, CO 80045, USA; paul.rozance@cuanschutz.edu

**Keywords:** placenta, glucose uptake, SLC2A3, transcriptomics, insulin, glucagon

## Abstract

Glucose, the primary energy substrate for fetal oxidative processes and growth, is transferred from maternal to fetal circulation down a concentration gradient by placental facilitative glucose transporters. In sheep, SLC2A1 and SLC2A3 are the primary transporters available in the placental epithelium, with SLC2A3 located on the maternal-facing apical trophoblast membrane and SLC2A1 located on the fetal-facing basolateral trophoblast membrane. We have previously reported that impaired placental SLC2A3 glucose transport resulted in smaller, hypoglycemic fetuses with reduced umbilical artery insulin and glucagon concentrations, in addition to diminished pancreas weights. These findings led us to subject RNA derived from *SLC2A3*-RNAi (RNA interference) and NTS-RNAi (non-targeting sequence) fetal pancreases to qPCR followed by transcriptomic analysis. We identified a total of 771 differentially expressed genes (DEGs). Upregulated pathways were associated with fat digestion and absorption, particularly fatty acid transport, lipid metabolism, and cholesterol biosynthesis, suggesting a potential switch in energetic substrates due to hypoglycemia. Pathways related to molecular transport and cell signaling in addition to pathways influencing growth and metabolism of the developing pancreas were also impacted. A few genes directly related to gluconeogenesis were also differentially expressed. Our results suggest that fetal hypoglycemia during the first half of gestation impacts fetal pancreas development and function that is not limited to β cell activity.

## 1. Introduction

Functional placental insufficiency is a major cause of intrauterine growth restriction (IUGR), which commonly results in hypoglycemic fetuses due to reduced placental transfer of glucose [1,2,3]. Sheep models of IUGR have been able to recapitulate many of the same challenges that human pregnancies with IUGR experience, particularly fetal hypoglycemia [2,3,4]. Glucose, the primary energy substrate for fetal and placental oxidative processes and growth, is not produced endogenously until near term and therefore relies on transfer from the maternal circulation. In sheep, this is accomplished through a maternal-to-fetal glucose concentration gradient and facilitative glucose transporter proteins on both maternal-facing apical microvillus SLC2A3 (GLUT3) and fetal-facing basal trophoblast membranes SLC2A1 (GLUT1; [5]). The distinct localization of these two glucose transporters makes the sheep placenta an ideal model for studying the relative importance of apical versus basolateral glucose transport.

The transport of glucose from the maternal circulation plays a crucial role across different stages of pancreatic development. During the primary transition period (before 24 days gestational age [dGA] in sheep; 25–26 dGA in humans), glucose is needed for early organogenesis, endocrine cell development (including early insulin expression), and proliferation of multipotent progenitor cells, which have been shown to directly contribute to the mature size of the pancreas [6].

During the second transition period (approximately 20% of gestation to term) is when isletogenesis, branching, and full differentiation of β- and α-cells occur, including the start of β-cell responsiveness to glucose [7]. All of these are essential processes to prepare the fetus for the regulation of glucose metabolism later in pregnancy and into adult life. Perturbed glucose transport across gestation can therefore result in compounding insults to pancreatic growth and function. Studies of IUGR’s impacts on the fetal pancreas in multiple species have demonstrated inappropriate insulin responses to glucose, likely due to pancreatic insufficiency from β-cell deficiency [2] and a global reduction in pancreatic endocrine tissue mass [8,9].

Our laboratory has previously published a sheep model of diminished placental SLC2A3 glucose transport at mid-gestation (75 dGA) using in vivo lentiviral-mediated RNA interference (RNAi; [10]), which resulted in smaller, hypoglycemic fetuses with reduced umbilical artery insulin and glucagon concentrations, in addition to diminished pancreas weights. These findings lead us to subject RNA derived from fetal pancreases from both *SLC2A3*-RNAi and NTS-RNAi pregnancies to real-time quantitative PCR (qPCR) and transcriptomic analysis to further elucidate the impact of placental SLC2A3 deficiency on fetal metabolism. We hypothesized that the impact of hypoglycemia on the fetal pancreas will have a global impact on genes related to growth and function as well as a direct impact on mRNA expression of glucagon and insulin.

## 2. Results

### 2.1. Quantitative Real-Time PCR

**Figure 1:**
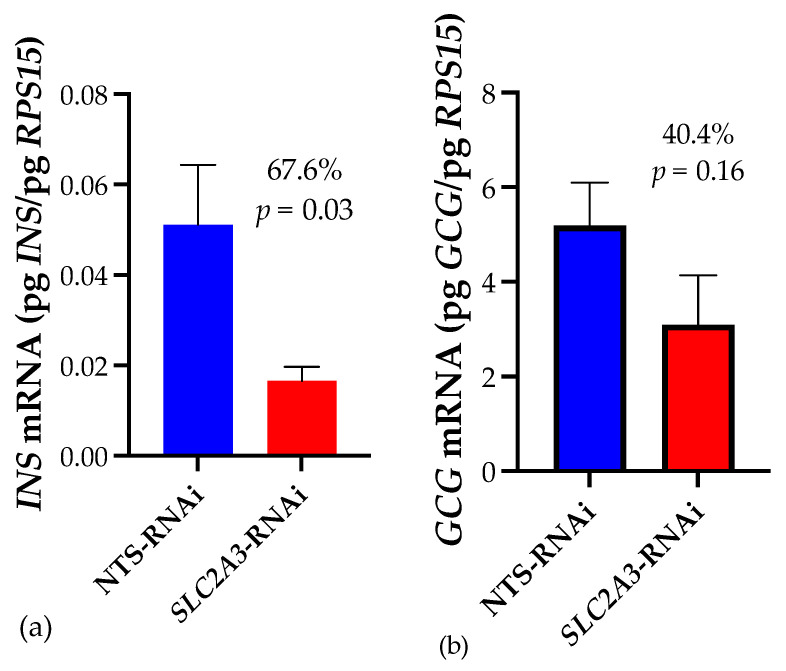
Impact of *SLC2A3*-RNAi on fetal pancreatic concentrations of (**a**) *INS* and (**b**) *GCG* mRNA. Data are shown as means ± SEM. NTS, non-targeting sequence; RNAi, RNA interference.

Fetal pancreas insulin mRNA concentrations (Figure 1) were 67.6% lower in *SLC2A3*-RNAi pregnancies (*p* ≤ 0.05), whereas there was a 40.4% difference in glucagon mRNA, although this was not statistically different.

### 2.2. Transcriptomic Analysis

**Figure 2:**
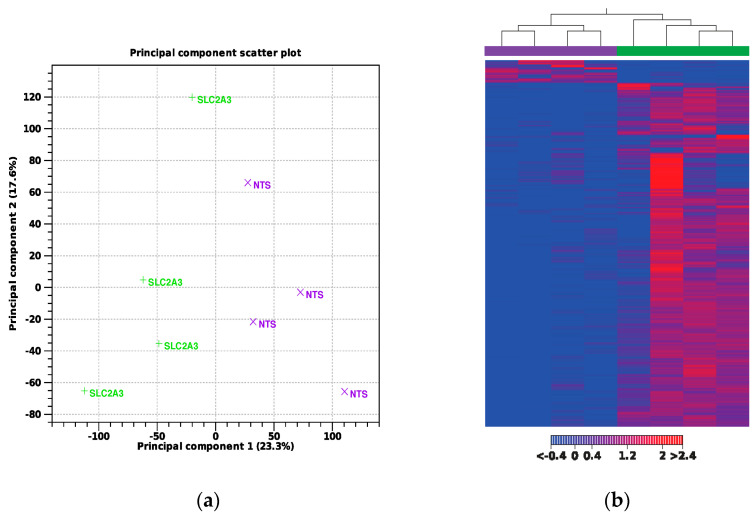
(**a**) Principal component analysis and (**b**) hierarchical heat map. Samples in purple denote NTS-RNAi, while green denotes *SLC2A3*-RNAi.

**Figure 3:**
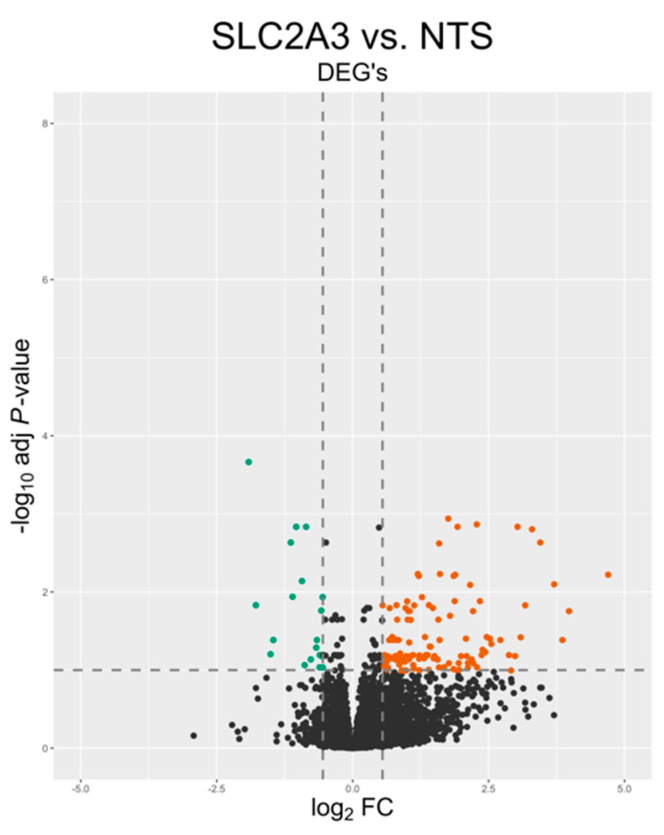
Volcano plot displaying differentially expressed genes (DEGs). Green denotes downregulated and red denotes upregulated. Black dots represent transcripts that were not significantly differentially expressed.

A total of *n* = 4 samples per group were used for transcriptomic analysis. Approximately 157 million reads were sequenced from both groups. After adapter trimming and quality filtering, 100% of reads were retained. An average of 98.2% of the quality-controlled (QC) reads were mapped to the sheep reference genome. A summary of the total number of reads and the mapping results for each sample is presented in Table 1. Principal component analysis (PCA) and a hierarchical heat map (Figure 2) revealed samples from the two treatments grouped into two distinct clusters. A total of 771 genes met our thresholds of a false discovery rate (FDR) of Q ≤ 0.1 and absolute fold change (FC) of >1.5, with 714 being upregulated and 57 being downregulated, which is illustrated in the volcano plot in Figure 3. Notable DEGs are featured in Table 2. Due to few downregulated genes, gene ontological (GO) enrichment analysis revealed predominantly pathways affected by gene upregulation.

Biological processes (BPs) identified by the GOTERM_BP_DIRECT database included pathways associated with multiple metabolic processes, gluconeogenesis, digestion, and negative regulation of cell proliferation (Table 3). Twelve (17%) genes contributing to biological processes overlapped with more than one biological process.

**Figure 4:**
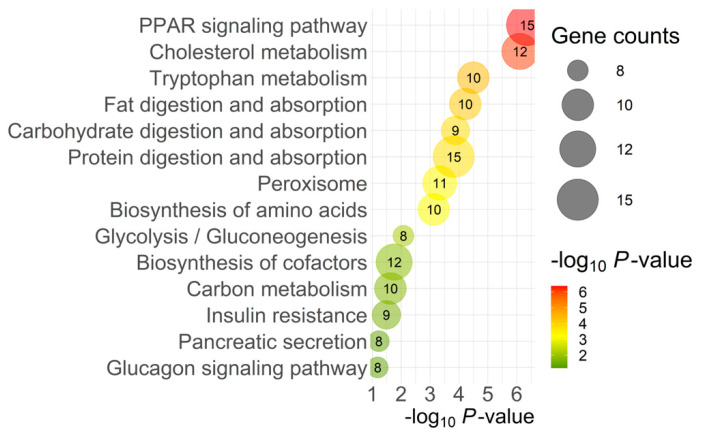
Top KEGG pathways from DAVID analysis. The color and size of each bubble represent the *p*-value and number of gene counts.

The top pathways identified by KEGG analysis of DEGs were predominantly related to metabolism (96 genes). Figure 4 presents the specific metabolic pathways identified by KEGG pathway analysis, excluding the overall metabolism category.

**Figure 5:**
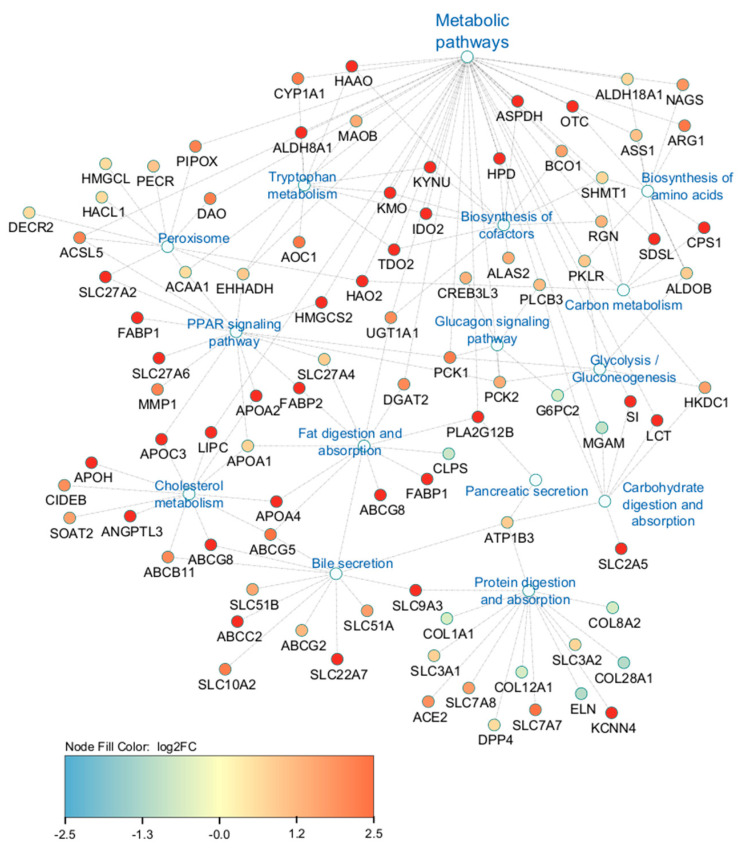
Cytoscape interactive network of DEGs involved in highly enriched pathways related to metabolism.

The interaction networks of DEGs involved in highly enriched pathways are presented in Figure 5, which demonstrates the interaction between genes from the different enriched pathways and their relation to the large number of genes contributing to the metabolic pathways term. Of the 245 genes contributing to the interaction networks in Figure 5, 37% (91 genes) had overlapping contributions to more than one enriched pathway.

## 3. Discussion

Glucose is the primary energy substrate for fetal and placental development and growth [7]. Its delivery to the fetus is reliant upon the maternal-to-fetal concentration gradient enabled by facilitative glucose transporters. In sheep, the locations of SLC2A3 and SLC2A1 on the trophoblast membranes create an ideal model for investigating the relative importance of apical (SLC2A3) compared to basolateral (SLC2A1) glucose transport [5]. The importance of placental glucose uptake, transport, and metabolism is highlighted in IUGR pregnancies, in which the degree of fetal hypoglycemia correlates with the severity of IUGR [1,11]. Glucose availability also plays a crucial role in different stages of pancreatic development. During the primary transition period, glucose is essential for organogenesis, endocrine cell development, and the proliferation of multipotent progenitor cells. The second transition period sees the differentiation of β- and α-cells and the beginning of β-cell responsiveness to glucose, setting the stage for the regulation of glucose metabolism later into pregnancy and postnatal life [12,13].

We previously established [10] that diminished placental SLC2A3 concentrations during the first-half of gestation results in smaller, hypoglycemic fetuses that exhibited reduced umbilical artery insulin and glucagon concentrations, and smaller pancreases. This led us to hypothesize that those pancreases were enduring a global effect of hypoglycemia on development and growth, not simply impaired β-cell function. Our current results of a 67.6% reduction in insulin mRNA and 40.4% reduction in glucagon mRNA in *SLC2A3-*RNAi fetal pancreases (Figure 1) align with the reported reductions in those hormones in umbilical arterial blood [10]. Reduced insulin secretion, smaller pancreases, and pancreatic dysfunction have all been shown to be hallmarks of the IUGR fetus [12]. A sheep model of near-term IUGR demonstrated impaired insulin secretion as a result of significantly reduced insulin content of the fetal pancreases, with deficient insulin storage and/or biosynthesis, in addition to decreased β-cell mass [2]. Other sheep models demonstrate hypoglycemic fetuses’ insulin secretion is diminished despite similar numbers of β-cells, that did not fully recover even when glucose was returned to normal concentrations, revealing hypoglycemic programming of fetal β-cell function [6]. Chronic hypoglycemia in similar studies have bolstered this β-cell programming effect in addition to decreased β-cell mass and impaired glucose-stimulated insulin secretion (GSIS; [12]). Clearly, the smaller pancreases observed in the hypoglycemic, *SLC2A3*-RNAi fetuses could have smaller β-cell masses and therefore the ability to secret insulin at sufficient levels.

Therefore, we conducted a transcriptomic analysis of fetal pancreases from *SLC2A3*- and NTS-RNAi pregnancies to further investigate how fetal hypoglycemia during the first-half of gestation contributed to impaired pancreatic growth and function. Our findings demonstrate impacts on pathways related to the critical transition periods of pancreatic development during early- to mid-gestation, including both organogenesis and pancreatic function, and altered pancreatic metabolism, with potential long-term impacts on glucose homeostasis. In the context of early pregnancy and pancreatic organogenesis, our analysis revealed several pathways associated with cellular signaling and growth. *SHH*, an important ligand in hedgehog signaling, was significantly upregulated (log2FC = 1.399). Hedgehog signaling regulates growth, differentiation, and function in many organs in both the fetus and adult; however, in the pancreas, *SHH*’s role in pancreatic development is more complex. Studies from mice have demonstrated that increased hedgehog signaling antagonizes organogenesis, and *SHH-*specific inhibition is a prerequisite for pancreas formation, with other hedgehog ligands (i.e., *IHH*) likely promoting pancreatic formation [14,15]. Upregulation of *SHH* in the *SLC2A3*-RNAi fetal pancreas fits with these studies in mice and could help explain the reduced pancreas size and initial organogenesis during fetal hypoglycemia [10].

The hepatocyte nuclear factor (*HNF*) family of transcription factors has been implicated in the early stages of organ formation, including interacting with crucial pancreatic transcription factors such as *PDX1*, a marker of pancreatic progenitor cells critical to β-cell determination [13,16,17]. Hepatocyte Nuclear Factor 4 Alpha (*HNF4α*) was differentially expressed (log2FC = −0.599) and, in the analysis of biological processes, contributed to pathways associated with the negative regulation of cell proliferation as well as phospholipid and cholesterol homeostasis. A study investigating transcriptional regulatory networks of pancreatic islets found that *HNF4α* contributes to a large fraction of pancreatic islet transcriptomes by binding directly to nearly half of the actively transcribed genes [18]. *HNF4α*’s large influence on organogenesis and the development of functional β-cells has also been shown in many mouse studies. One such study, by Chen et al. [19], demonstrated that homozygous germline ablation of *HNF4α* is embryonically lethal, even before pancreas development. However, β-cell specific ablation of *HNF4α* resulted in hyperinsulinemia in utero and reduced blood glucose levels at birth [20]. Additionally, mutation of *HNF4α* in people is associated with MODY (maturity onset diabetes of the young; [13,18,20]), further demonstrating its crucial contribution to glucose homeostasis. These studies highlight the broad influence of *HNF4α* on the pancreatic transcriptome and could explain why changes in its expression, specifically the downregulation seen in our study, could have such a large impact on both the growth and function of the fetal pancreas.

The secondary transition period of the fetal pancreas overlaps with the first transition period in both sheep and humans, and occurs over a broad period from around 20% of gestation to term [12,21]. It is characterized by growth, branching, and differentiation into the different pancreatic cell lineages that coincide with an increase in endocrine and exocrine gene expression [13]. In our model, many genes and pathways specifically associated with pancreatic function were affected, in addition to metabolism and delivery of energetic substrates. KEGG pathways analysis revealed several genes contributing to pancreatic secretion: *PLCB3*, *PLA2G12B*, *CLCA1*, *ATP1B3*, *ITPR3*, *CCK*, *ATP2B1*, *SLC26A3*, some of which overlap with other biological processes. For example, *PLCB3* and *PLA2G12B* both contribute to lipid catabolic processes, demonstrating the complex interactions between intracellular signaling and pancreatic function. Arguably the most notable DEG in this list is *CCK*, which was upregulated (log2FC = 2.852). *CCK* acts on numerous tissues in the body but is primarily involved in metabolism and digestion. It is best known for the stimulation of exocrine pancreas enzyme secretion but has also been shown to stimulate adaptive pancreatic growth in neonatal and adult rodent models [22]. Little research exists on the role of CCK during pancreatic development, especially in sheep; however, studies in mice have shown the expression of *CCK* as a marker of endocrine cell lineage early on, as well as expression in acinar and α-cells in later stages [23]. A study utilizing human pancreatic tissues reported that CCK-B/Gastrin receptors mediate the autocrine effects of gastrin on developing islets in the fetal pancreas, implicating an important role for *CCK* in the regulation of glucose homeostasis [24].

Another pathway highlighted by KEGG analysis related to intercellular signaling was Peroxisome Proliferator-Activated Receptor (PPAR) signaling. PPAR signaling is involved in lipid homeostasis during development throughout the body, and disruption of PPARs has been linked to metabolic diseases, such as lipid accumulation in pancreatic beta cells with implications for diabetes [25]. In zebrafish development, disruption of PPAR signaling results in ligand-specific impaired endocrine and exocrine pancreas development and disrupted gene expression of key developmental genes for the pancreas (i.e., *PDX1*; [25]). Additionally, PPARβ/δ knockout mice have impaired insulin secretion [26]. In our model, multiple genes related to peroxisomes and PPAR signaling were upregulated (Figure 4), including some overlapping contributions from *ACAA1* (Acetyl-CoA acyltransferase 1), *EHHADH* (enoyl-CoA hydratase and 3-hydroxyacyl CoA dehydrogenase), and *SLC27A2*. *ACAA1* and *EHHADH* are both involved in β-oxidation throughout the body and likely contribute to lipid metabolism within the pancreas, in concert with *SLC27A2*’s role in fatty acid transport. This further demonstrates the complexity and interconnectedness of intercellular signaling and substrate transport with pancreatic growth, metabolism, and function.

Many biological processes (Table 2) related to lipid metabolism were affected, including phospholipid homeostasis, lipid catabolic processes, fatty acid metabolic processes, and transport. KEGG pathways analysis (Figure 4) also revealed many genes contributing to fat digestion and absorption pathways. Free fatty acids (FFAs) in particular have complex actions in the pancreas, but nevertheless are important regulators in glucose metabolism and insulin release, and the activation of their receptors directly impacts pancreas secretion [27]. They can also stimulate the release of CCK from enteroendocrine cells, which in turn induces pancreatic enzyme secretion. While short-term exposure to FFAs, specifically long-chain fatty acids, results in insulin release from β-cells, it has been well documented that chronic exposure and/or lipotoxicity results in desensitization, impaired GSIS, insulin resistance, and, over the long-term, β-cell damage [27,28,29,30].

Fatty acids are also oxidized by the TCA cycle within the mitochondria, yielding ATP, which can contribute to increased ATP concentrations, plasma membrane depolarization via ATP-sensitive K+ channels (K_ATP_), and eventual Ca^2+^ dependent exocytosis of insulin [29,31,32]. Pancreases from *SLC2A3*-RNAi fetuses had increased expression of members of the solute carrier family 27 (*SLC27A2*, *SLC27A4, SLC27A6*), all of which are long-chain fatty acid transport transmembrane proteins [31]. Upregulation of these transporters could be a compensation of the hypoglycemic fetal pancreas to increase intake of fatty acids for use as an energetic substrate and also an attempt to maintain insulin responsiveness, albeit at lower levels than their NTS-RNAi counterparts. FFA uptake, storage, and oxidation can also be regulated by binding to PPARs within the cell [33]. PPAR signaling also maintains lipid metabolic homeostasis during cold stress with reduced plasma glucose concentrations in fish [34].

In our analysis of biological pathways, the term “negative regulation of cell proliferation” contains one of the few downregulated genes: *ST18*. While not much is known about *ST18*’s role in the body and particularly pancreatic development, Henry et al. [35] investigated *ST18*’s expression in the developing mouse pancreas, demonstrating its expression specific to endocrine cells. They also observed increased expression in the presence of cytotoxic levels of free fatty acids, implicating *ST18*’s role as a transcriptional regulator of lipotoxicity and cytokine-induced β-cell apoptosis, and therefore impaired insulin secretion. It is possible that the downregulation of *ST18* coupled with the increased expression of pathways related to lipid metabolism and fatty acid metabolic processes seen in our data demonstrate a shift in energetic substrates from glucose to lipids that is just beginning by mid-gestation and has not reached the lipotoxic levels that Henry et al.’s [35] study achieved in their goal of emulating the environment of type 2 diabetes. Regardless, while there remains a large knowledge gap in the biological function of *ST18*, it appears to have a clear role in β-cell mass and function.

Finally, KEGG pathways analysis also revealed pathways related directly to pancreatic function, namely pancreatic secretion, insulin resistance, glycolysis, gluconeogenesis, and the glucagon signaling pathway. Many of the DEGs involved overlapped in their contributions to those pathways, making it difficult to interpret which processes were most impacted; however, the genes involved demonstrate a clear impact on the functionality of the pancreas. Many of these encode essential regulatory enzymes in gluconeogenesis. For example, *PCK1* is the cytosolic form of phosphoenolpyruvate carboxykinase and *PCK2* the mitochondrial form; both catalyze the formation of phosphoenolpyruvate from oxaloacetate, an essential step in gluconeogenesis. *FBP1* catalyzes the hydrolysis of fructose 1,6-bisphosphate to fructose 6-phosphate. *PCK1*, *PCK2*, and *FBP1*, when deficient, have all been associated with hypoglycemia [36], and may have been upregulated as a compensatory mechanism to potentially provide needed intermediates within the glycolytic pathway.

One of the few genes that were downregulated includes *G6PC2*, which is involved in the final step of gluconeogenesis. *G6PC2* (glucose-6-phosphatase catalytic subunit 2) is islet-specific, and studies of G*6PC2*^−/−^ mice confirm its essential role in glycolytic flux and sensitivity of GSIS. Isolated islets from *G6PC2*^−/−^ mice exhibit a left shift in GSIS, such that under fasting conditions, insulin levels are the same as wild-type mice, inferring *G6PC2*’s potential role in protecting against hypoglycemia under stressed conditions [37]. A more recent study utilizing the same model also implicates *G6PC2* as a negative regulator of oxidative metabolism in the TCA cycle [38]. In our model, *G6PC2* was downregulated in response to hypoglycemia and could contribute to a potentially altered GSIS response in our fetuses.

Fetal hypoglycemia during the first half of gestation, generated by placenta-specific *SLC2A3*-RNAi [10], altered the fetal pancreas transcriptome in multiple aspects. Many interconnected pathways contributing to pancreatic function and glucose metabolism were affected by *SLC2A3*-RNAi, demonstrating a multifaceted and broad impact of hypoglycemia on the fetal pancreas that is challenging to interpret. However, the smaller size of the *SLC2A3*-RNAi pancreases is impacting fetal metabolism, as evidenced by the reduced expression of *INS* and *GCG* mRNA, and reduced umbilical concentrations of INS and GCG [10]. Our data implicate altered pathways directly related to both pancreatic transition periods needed for growth and metabolism as well as pancreatic function. The upregulation of genes involved with fatty acid and lipid metabolism may suggest fatty acid utilization as compensatory energy sources for development and metabolism by the pancreas in early fetal life.

## 4. Materials and Methods

All procedures conducted with animals were approved by the Colorado State University Institutional Animal Care and Use Committee (Protocol 1483), as well as the Institutional Biosafety Committee (17-039B).

### 4.1. Generation of Lentivirus and SLC2A3-RNAi Pregnancies

Lentiviral generation and titering of *SLC2A3-*RNAi and NTS-RNAi were conducted as described previously [39], with the shRNA sequences for *SLC2A3*-RNAi and NTS-RNAi constructs previously reported by Lynch et al. [10]. Animal management, estrus synchronization, embryo transfers, and the generation of *SLC2A3-*RNAi and NTS-RNAi pregnancies were performed as previously described [4,10,39]. In summary, all ewes (Dorper breed composition) were group-housed in pens at the Colorado State University Animal Reproduction and Biotechnology Laboratory, and were provided access to hay, trace minerals, and water to meet or slightly exceed their National Research Council [40] requirements. Lentiviral infection of day-9-hatched sheep blastocysts was used to stably integrate and express shRNA targeting *SLC2A3* mRNA or a non-targeting sequence in the trophectoderm. Tissues from 6 NTS-RNAi (5 males and 1 female) and 6 *SLC2A3*-RNAi (3 males and 3 females) pregnancies were collected during terminal surgeries at 75 dGA [10].

### 4.2. RNA Isolation

Frozen fetal pancreases were pulverized, and RNA was isolated using the RNeasy Mini Kit (QIAGEN, Hilden, Germany), according to the manufacturer’s instructions. RNA concentration was quantified using the BioTek Synergy 2 Microplate Reader (BioTek, Winooski, VT, USA). RNA quality was measured by the 260/280 nm absorbance ratio, and RNA samples were stored at −80 °C until use.

### 4.3. cDNA Synthesis and Quantitative Real-Time PCR

cDNA was generated from 2 µg of total cellular RNA using iScript Reverse Transcription Supermix (BioRad, Hercules, CA, USA according to the manufacturer’s protocol, with quality measured by the 260/280 absorbance ratio. An equal mass of cDNA (10 ng/µL) was used for each sample in the quantitative real-time PCR (qPCR) reaction. qPCR was performed using the CFX384 Real-Time System (BioRad). Forward and reverse primers for qPCR were designed using NCBI’s Primer-BLAST tool [41] to amplify an intron-spanning product. Primer sequences and amplicon size are summarized in Table 4. Standard curves were generated as described previously [42]. Briefly, a PCR product for each gene was generated using cDNA from pooled 75 dGA fetal pancreases as a template and cloned into the StrataClone vector (Agilent Technologies, Santa Clara, CA, USA), and each PCR product was sequenced to verify amplification of the correct cDNA. Using the PCR products amplified from the sequenced plasmids, standard curves were generated for each mRNA from 1 × 10^2^ to 1 × 10^−5^ pg and were used to measure amplification efficiency. The starting quantity (pg) was normalized by dividing the starting quantity of mRNA of interest by the starting mRNA quantity (pg) of ribosomal protein S15 (RPS15; [42]). Normalized values were compared using the unpaired Student’s *t*-test, with *p* ≤ 0.05 considered significant and a trend at *p* ≤ 0.10.

### 4.4. RNA Sequencing, Alignments, and Analysis

Assessment of the RNA samples’ integrity and quality, library generation, and RNA-Seq (2 × 150 cycles, 80 million paired-end reads/sample; NovSeq6000, Illumina, San Diego, CA, USA) was conducted by the Genomics Shared Resource Core Facility, University of Colorado Anschutz Medical Campus (Aurora, CO, USA). The sequencing data from this study have been deposited in NCBI’s Gene Expression Omnibus [43] and are accessible through GEO Series accession number GSE261932 (https://www.ncbi.nlm.nih.gov/geo/query/acc.cgi?acc=GSE261932; available April 26, 2024).

RNA integrity was assessed by Agilent TapeStation (Agilent Technologies, Inc., Santa Clara, CA, USA) and all samples had a minimum RNA integrity number (RIN) of ≥9.0. RNAseq analysis was performed on the Illumina NovaSEQ6000 (Illumina, San Diego, CA, USA) platform (100,000,000 paired end reads) and FASTQ files were generated for each sample. Data were assessed for quality with FastQC tool version 0.11.9 (Babraham Bioinformatics, Babraham Institute, Babraham, England), as well as with QIAGEN CLC Genomics Workbench software (QIAGEN, Hilden, Germany), version 21 (https://www.qiagenbioinformatics.com). CLC Genomics workbench was then used for trimming of raw sequencing reads based on quality score (Q-score > 30), read length (≥15 nucleotides), and removal of adapter sequences. Raw sequence reads were mapped to the reference ovine genome (*Ovis aries*, Oar.ra_1.0, Baylor College of Medicine, Houston, TX, USA) with default CLC Genomics Workbench software parameters applied. Expression data were normalized using the trimmed mean of M-values (TMM) normalization method [44] and presented as fragments per kilobase of transcript per million mapped reads (FPKM).

Differential expression analysis for *n* = 4 sample per group was performed using the CLC Genomics Workbench Differential Expression tool as well as the DESeq2 package [45] in R studio (RStudio Team (2022), RStudio: Integrated Development for R. RStudio, PBC, Boston, MA, USA, http://www.rstudio.com/) for comparison. Thresholds for differentially expressed genes were a false discovery rate of Q ≤ 0.1 and absolute fold-change of >1.5. Functional annotation of DEGs was performed using the Database for Annotation, Visualization, and Integration Discovery (DAVID, https://david.ncifcrf.gov/). Pathways were determined from the KEGG database [46] and biological processes from GOTERM_BP_DIRECT annotation sets. Interaction networks were constructed with the Cytoscape software version 3.10.2 [47]. Finally, additional insight into altered networks was obtained by submitting the same list of DEGs to QIAGEN Ingenuity Pathway Analysis version 111725566 ([48], QIAGEN, Hilden, Germany, https://digitalinsights.qiagen.com/IPA). The threshold for considering a pathway significant was *p* ≤ 0.05.

## 5. Conclusions

The results of our transcriptomic analysis indicate that fetal hypoglycemia had a generalized impact on pancreas growth and development, identifying pathways involved in growth and metabolism by the pancreas, spanning both transition periods. Of note are altered expression of genes involved in carbohydrate and fatty acid metabolism, suggesting a potential compensatory shift towards a greater utilization of fatty acids for metabolism. If a true compensatory shift in substrate utilization occurred, this could have long-term impacts on pancreas function. The true impact of the altered fetal pancreas transcriptome at mid-gestation, as the result of *SLC2A3*-RNAi-induced fetal hypoglycemia, awaits thorough physiological assessment later in gestation or postnatally.

## Figures and Tables

**Table 1:** Summary of sequence reads mapped to sheep reference genome.

Group	Sample I.D.	Raw Reads	Quality Control (QC) Reads	Mapping Rate (%)
*SLC2A3*-RNAi	2_472	201,264,985.3	185,063,154	98.26
	3_472	175,336,416.6	160,783,494	98.06
	5_472	190,077,663.5	175,232,598	98.24
	6_472	192,195,658	176,704,688	98.21
NTS-RNAi	7_SC	169,767,017.4	155,846,122	98.27
	8_SC	179,660,289.9	164,874,248	98.11
	11_SC	129,489,997.8	118,975,410	98.22
	12_SC	162,587,201.6	149,141,240	98.19

**Table 2:** Notable differentially expressed genes (DEGs).

Gene	Name	Category	Log2FC	FDR
*CLPS*	Colipase	Fat digestion and absorption	−0.80984	0.000412
*CCK*	Cholecystokinin	Pancreatic secretion	2.85173	2.48 × 10^−13^
*SHH*	Sonic Hedgehog Signaling Molecule	Cellular development/growth and proliferation; tissue development	1.39917	0.023658
*PLCB3*	Phospholipase C Beta 3	IP3-DAG signaling	1.17916	4.17 × 10^−12^
*SLC26A3*	Solute Carrier Family 26 Member 3	Ion transport; pancreatic secretion	2.57893	4.02 × 10^−9^
*SLC27A2*	Solute Carrier Family 27	PPAR signaling; fatty acid transport	2.60986	3.77 × 10^−10^
*SLC27A4*	Solute Carrier Family 27	PPAR signaling; fatty acid transport	0.8981	0.00025893
*TDO2*	Tryptophan 2,3-dioxygenase	Tryptophan metabolism	4.23593	0.0088385
*HAA0*	Hydroxyacid Oxidase	Tryptophan metabolism; biosynthesis of cofactors	3.13823	7.24 × 10^−13^
*KYNU*	Kynureninase	Tryptophan metabolism; biosynthesis of cofactors	2.62505	1.02 × 10^−7^
*HNF4A*	Hepatocyte Nuclear Factor 4 Alpha	Negative regulation of cell proliferation	0.58768	0.00751424
*ST18*	ST18 C2H2C-type zinc finger transcription factor	Negative regulation of cell proliferation; apoptosis	−0.59897	0.00458748
*HS3ST1*	Heparan sulfate-glucosamine 3-sulfotransferase 1	Cell signaling; growth factor binding; tissue development	0.88156	0.07424441
*CREB3L3*	cAMP Responsive Element Binding Protein 3 Like 3	Glucagon signaling; insulin resistance; pancreatic secretion	1.3806	8.09 × 10^−12^
*FBP1*	Fructose-bisphosphatase 1	Glucagon signaling; carbon metabolism; glycolysis/gluconeogenesis	0.6707	0.0013089
*LDHA*	Lactate dehydrogenase A	Glucagon signaling; glycolysis/gluconeogenesis	0.73	0.00382388
*PCK1; PCK2*	Phosphoenolpyruvate carboxykinase 1; 2	Glucagon signaling; insulin resistance; glycolysis/gluconeogenesis; PPAR signaling	2.2652; 1.4892	1.5541 × 10^−7^; 2.31 × 10^−18^
*G6PC2*	Islet specific glucose-6-phosphatase catalytic subunit 2	Glucagon signaling; insulin resistance; glycolysis/gluconeogenesis; carbohydrate digestion	−0.6281	0.02096731

**Table 3:** GOTERM_BP_DIRECT biological processes.

Term	Count	*p*-Value	Genes	Fold Enrichment	FDR
Carbohydrate metabolic process	10	0.00353	*LDHA*, *MGAM*, *GNPDA1*, *SI*, *SLC3A1*, *SLC3A2*, *NPL*, *RENBP*, *FBP1*, *GLB1L3*	3.26251	0.44330
Cholesterol homeostasis	10	0.00003	*EHD1*, *ABCG8*, *ABCG5*, *DGAT2*, *LIPC*, *SOAT2*, *HNF4A*, *APOA2*, *ANGPTL3*, *ABCB11*	6.24377	0.03353
Negative regulation of cell proliferation	10	0.08406	*SLC9A3R1*, *HNF4A*, *CLDN19*, *PTK2B*, *PTH1R*, *PODN*, *ST18*, *SKAP2*, *ENPP7*, *BMP5*	1.88614	0.99922
Cell surface receptor signaling pathway	6	0.05824	*VIPR1*, *ADGRG7*, *EDN3*, *GLP2R*, *PTH1R*, *F2*	2.85899	0.99922
Lipid catabolic process	6	0.05054	*PLCB3*, *LIPC*, *PLA2G12B*, *CLPS*, *NEU1*, *PLBD1*	2.97648	0.99922
Cholesterol metabolic process	5	0.01663	*CUBN*, *LIPC*, *SOAT2*, *APOA2*, *ANGPTL3*	5.02971	0.97370
Fatty acid metabolic process	5	0.01510	*NAAA*, *CYP1A1*, *ANGPTL3*, *HACL1*, *ABCB11*	5.17341	0.92632
Gluconeogenesis	4	0.00888	*G6PC2*, *PCK1*, *FBP1*, *PCK2*	9.05347	0.71451
Phospholipid homeostasis	4	0.00379	*HNF4A*, *ANGPTL3*, *ITGB6*, *ABCB11*	12.07130	0.44330
Cell–cell signaling	3	0.08660	*SHH*, *GJB4*, *IHH*	6.03565	0.99922
Digestion	3	0.04159	*CLPS*, *CYM*, *AKR1D1*	9.05347	0.99922
Fatty acid transport	3	0.01449	*SLC27A6*, *SLC27A2*, *SLC27A4*	15.52024	0.92632

**Table 4:** Primers and product sizes for cDNA used in qPCR.

cDNA	Forward Primer (5′ → 3′)	Reverse Primer (5′ → 3′)	Product, bp
*RPS15*	ATCATTCTGCCCGAGATGGTG	TGCTTGACGGGCTTGTAGGTG	134
*INS*	GAGAGCGCGGCTTCTTCTAC	CGGGGCAGGTCTAGTTACAG	198
*GCG*	ACTCACAGGGCACATTCACC	CGGCGGAGTTCTTCAACGAT	274

## Data Availability

Data are available upon request to the corresponding author and through NCBI GEO Series accession number GSE261932.
